# Changes in the Radiocarbon Reservoir Age in Lake Xingyun, Southwestern China during the Holocene

**DOI:** 10.1371/journal.pone.0121532

**Published:** 2015-03-27

**Authors:** Aifeng Zhou, Yuxin He, Duo Wu, Xiaonan Zhang, Can Zhang, Zhonghui Liu, Junqing Yu

**Affiliations:** 1 MOE Key Laboratory of Western China’s Environmental Systems, Collaborative Innovation Centre for Arid Environments and Climate Change, Lanzhou University, Lanzhou, China; 2 Department of Earth Sciences, Zhejiang University, Hangzhou, China; 3 Department of Earth Sciences, The University of Hong Kong, Hong Kong Special Administrative Region, China; 4 Qinghai-Institute of Salt Lakes, China Academy of Sciences, Xining, China; University of Oxford, UNITED KINGDOM

## Abstract

Chronology is a necessary component of paleoclimatology. Radiocarbon dating plays a central role in determining the ages of geological samples younger than *ca*. 50 ka BP. However, there are many limitations for its application, including radiocarbon reservoir effects, which may cause incorrect chronology in many lakes. Here we demonstrate temporal changes in the radiocarbon reservoir age of Lake Xingyun, Southwestern China, where radiocarbon ages based on bulk organic matter have been reported in previous studies. Our new radiocarbon ages, determined from terrestrial plant macrofossils suggest that the radiocarbon reservoir age changed from 960 to 2200 years during the last 8500 cal a BP years. These changes to the reservoir effect were associated with inputs from either pre-aged organic carbon or ^14^C-depleted hard water in Lake Xingyun caused by hydrological change in the lake system. The radiocarbon reservoir age may in return be a good indicator for the carbon source in lake ecosystems and depositional environment.

## Introduction

The Indian Summer Monsoon domain is a key region to understand past climatic changes [1]. This region has continuous and well-preserved lake sediment, which provides excellent archives for paleoclimatic reconstructions of the terrestrial environment [1–3]. However, a critical task in paleolimnological studies is to establish a reliable age-depth model.

Many dating methods have been applied in lacustrine sediments, including ^210^Pb and ^137^Cs dating, optically stimulated luminescence (OSL) dating, and radiocarbon (^14^C) dating. Among these methods, radiocarbon dating has been used most extensively for providing a chronological framework for the past 50 kyr [4, 5]. Radiocarbon dating of sediment cores can be conducted on various materials, such as aquatic plant macrofossils, terrestrial plant macrofossils, and pollen grains [6–12]. Due to the lack of plant macrofossils or an insufficient concentration of pollen grains in lake sediments from barren landscapes [13, 14], bulk organic sediment and biogenic carbonate are usually chosen for radiocarbon dating instead. However, radiocarbon levels in bulk organic sediments might be "mixed" with fossil terrestrial carbon (old carbon) [15–17] and aquatic plants which draw its C from 14C-depleted water (i.e. because of the hard-water effect) [6, 9, 17]. These variables may produce a significant radiocarbon age difference between atmosphere and contemporaneous lake water system, which has been termed as "radiocarbon reservoir effect" [18]. Changes in reservoir age may be directly linked to changes in the hydrological setting (e.g. water inflow and outflow, gas exchange with the atmosphere, and the size of the carbon pool) and the variations in atmospheric ^14^C levels. As a consequence, reservoir ages might be a useful tool for reconstructing the response of lacustrine hydrology to climate change [19–23]. The uncertainties in dating lacustrine radiocarbon reservoirs significantly limit the application of radiocarbon dating in paleolimnological research, furthermore, hinder us from directly comparing among different paleoclimatic records. Anomalously large reservoir ages have been reported in many paleolimnological studies worldwide [14, 24–29].

In order to further understand the ^14^C reservoir effect, the following approaches have been applied [29]: modern calibration approach [30], linear extrapolation of ^14^C age model [31], geochemical modeling [13], stratigraphic alignment [32], and independent age determinations [29]. Nevertheless, the application of ^14^C dating to lake sediments is hindered by the choice of sampling materials which causes temporal and spatial variability because of the ^14^C reservoir effects [27, 33–35]. Although we can accurately evaluate the reservoir effect by measuring modern surface samples, the assumption that the reservoir age remains constant through time is not necessarily justified [29].

Although the radiocarbon reservoir effect of bulk organic matter from lakes has been recognized [18], many paleoclimatological studies continue to use chronologies derived from such materials because there is no alternative/better sampling material to date. In addition, the use of macrofossils of aquatic species (i.e. *Ruppia matitine*), which take their carbon from the lake water, for radiocarbon dating will also not provide reliable results due to the same reservoir issue [27]. An ideal radiocarbon chronology of the lake sediment sequence should be entirely based on the macrofossils of terrestrial plants [36]. Lake Xingyun is a great choice for the paleoclimatic study in southwestern China due to its location and its large catchment. The age profile is crucial for whether the relation between the Indian Summer Monsoonal strength and climates in Lake Xingyun during the Holocene can be posed. ^14^C age of bulk sediments have been measured from previous study [2], which has been cited for over 100 times without further conclusively justification on the age issue. Thus it is important and necessary to evaluate the reservoir effect in this lake and re-adjust the paleoclimatic view of this site/region. In this study, we examined the temporal variations of the ^14^C reservoir effect inLake Xingyun. As the ^14^C ages on bulk organic matter have been previously reported [2], here we use a new core collected from the middle of Lake Xingyun for comparison with the previous result. Tree twigs were carefully selected for ^14^C dating. Age profile of our new core and that of the previously reported were compared and the temporal variations in reservoir ages were assessed on the basis of the difference between the ages dated on different materials. Together with paleoclimatological results, we further discuss the relationship between reservoir age and hydrological change of this lake system. Finally, we re-evaluated the paleoclimatic interpretation of previous studies with additional evidence for a more convincing age model.

## Materials and Methods

**Fig 1:**
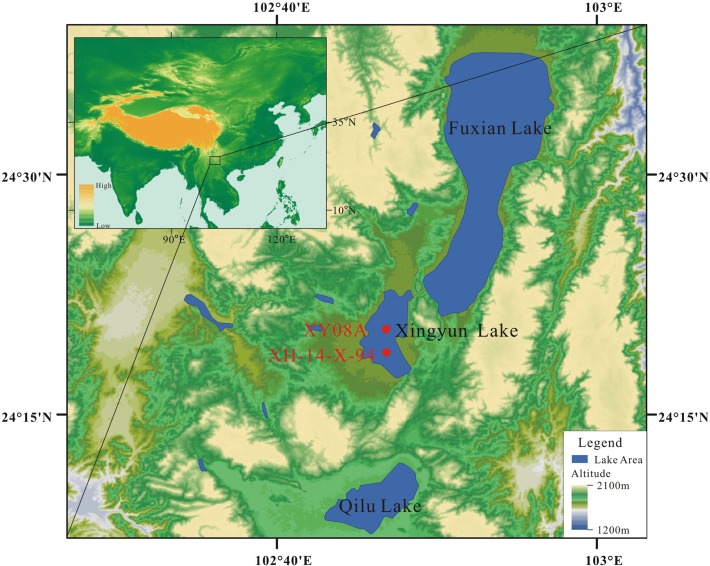
Map showing the topographical features, location and coring location XY08A, XH-14-X-94 of Lake Xingyun, SW China.

Lake Xingyun (24°17'20"N-24°23'05"N, 102°45'18"E-102°48'30"E, 1723 m a.s.l.), is a semi-closed shallow lake (maximum water depth 10.81m) located in the India Summer Monsoon (ISM) controlled area of Asia. The surface area is 34.7 km^2^ and the catchment area is 386 km^2^ (Fig. 1). Several small intermittent rivers flow into Lake Xingyun, and the lake itself drains northward via the Gehe River into Lake Fuxian. Lake Xingyun is a freshwater lake with conductivity of 344 mS/cm and pH in the range of 8.4–8.7 [2]. Within the drainage basin, the mean annual temperature is 15.6°C. A mean annual precipitation of approximately 880 mm accumulates annually, with 85 to 90% of the precipitation falling between May and October under the influence of strong monsoonal southwesterly winds originating in the Bay of Bengal and Southern Indian Ocean [37]. In winter, the N–NW winds bring dry air to this region with minimal rainfall from November to March. Monsoon circulation has been shown to strongly affect the amount and isotopic composition of precipitation in Kunming [38], which is about 60 km north of the lake. The local vegetation is mainly composed of agricultural crops and pine forests. On the surrounding mountains, drought-tolerant shrubs and grasslands dominate, while pine forests and broadleaved forests or shrubs consisting of evergreen and deciduous *Quercus* are distributed at relatively high altitudes. The bedrock of the Lake Xingyun catchment is largely composed of dolomite, sandstone, and basalt [39].

**Fig 2:**
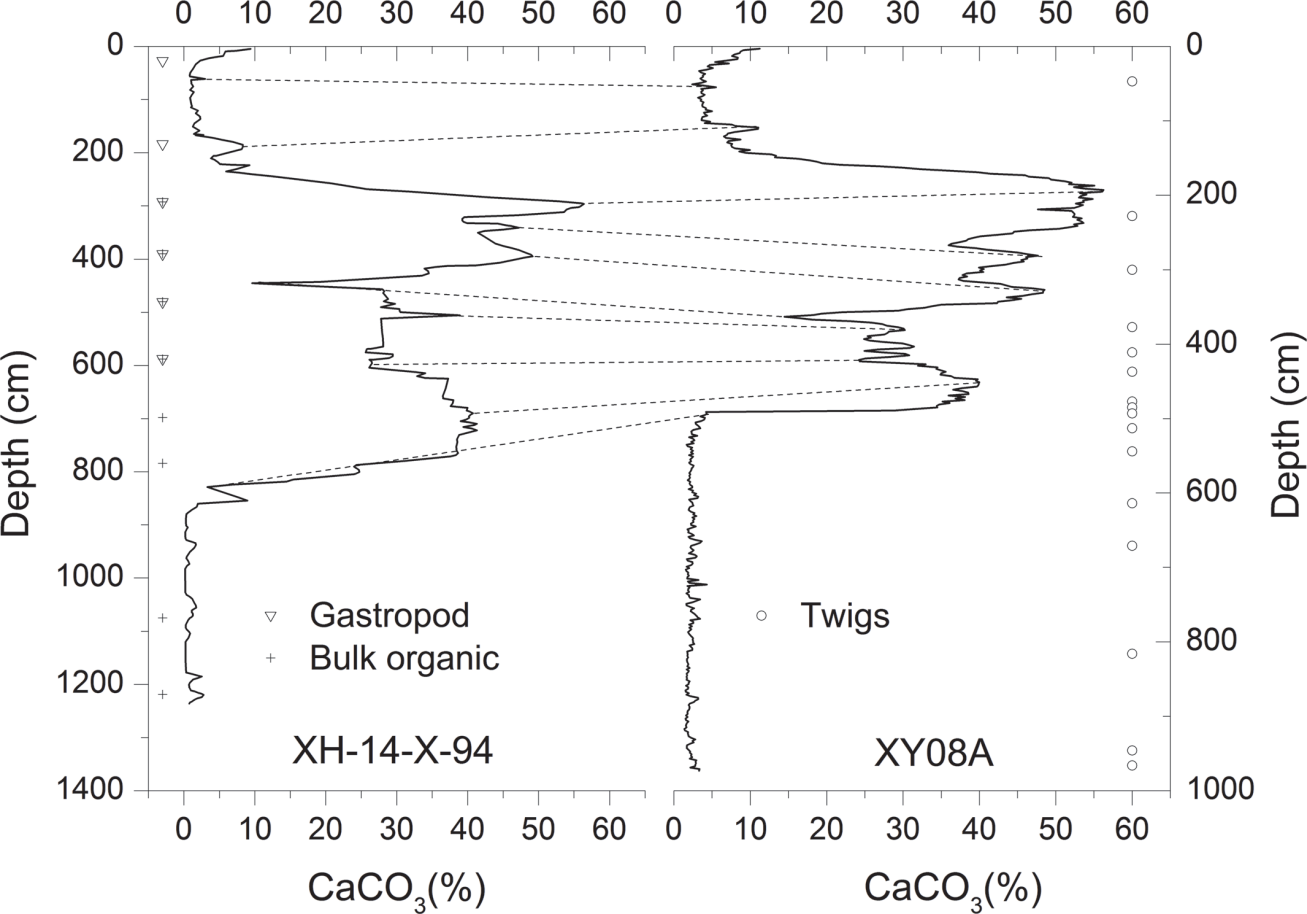
Comparison of core XH-14-X-94 [2] with core XY08A, Lake Xingyun, SW China.

Core XY08A (9.74 m long, Fig. 2) was collected at a water depth of 9.3 m from the central part of Lake Xingyun, using a UWITEC platform and piston corer set in October 2008. Core XY08A was sub-sampled at 2 cm intervals, freeze-dried and stored in a refrigerated room at 4°C. Core XY08A was mainly composed of silty clay and can be briefly described as follows: 490–980 cm, brown gray clay; 160–490 cm, grayish silty carbonate mud with saprogenic mud containing gastropod shells and plant remains; 0–160 cm, reddish silty clay.

The organic matter (OM) content was determined by loss on ignition (LOI) at 550°C in a muffle furnace for 5 hours, and the carbonate content was calculated by the mass loss at 950°C, assuming the loss of mass at 950°C was from decomposition of CaCO_3_ to CaO and CO_2_. The mass loss due to CO_2_ was multiplied by 2.27, the ratio of the molecular weights of CaCO_3_ and CO_2_ [40]. *n-*Alkane analyses were performed following the procedures described by He *et al*. [41]. Total lipids were extracted from freeze-dried sediments (*ca*. 2–6 g) with organic solvents (dichloromethane: methanol = 9:1, v/v). The neutral lipids were extracted with *n*-hexane. The *n*-alkanes were then further purified using silica gel column chromatography. The *n*-alkane fraction was analyzed using a Gas Chromatography Agilent 7890 equipped with a flame ionized detector at the University of Hong Kong, using *n*-C_36_ alkane for quantification and laboratory standards for peak identification.

A total of eight samples of terrestrial plant macrofossils were taken for ^14^C dating from different depths (Table 1). All of them were dated using accelerator mass spectrometry (AMS). Seven of the samples were dated at the AMS Dating Laboratory at Peking University, and one sample was dated at Beta Analytic, USA. All dates were calibrated to calendar years before the present (0 BP = 1950 AD) using the computer program CALIB Rev. 7.0 in conjunction with the IntCal13 calibration data set [5]. The age-depth model was established by fitting spline functions to the age controlling points using the Clam library [42, 43] implemented using the statistical software package with the default smoothing parameter of 0.3.

**Table 1:** Radiocarbon ages of bulk organic matter and plant remains in Lake Xingyun, SW China.

Laboratory number	Depth (cm)	Materials dated	δ^13^C (‰ VPDB)	^14^C age (yr BP)	Error (yr)	*Adjusted depth (cm)
OS-8168	183–184	Gastropod	-22.4	1620*	50	109
OS-9083	292–293	bulk organic	-27.6	3060	35	185
OS-9076	389–391	bulk organic	-28.64	4750	65	323
OS-9087	479–481	bulk organic	-28.18	6500	50	370.5
OS-9078	587–589	bulk organic	-28.2	7420	85	408.5
OS-9088	697–699	bulk organic	-28.01	8710	35	447
OS-9089	783–785	bulk organic	-29.52	9680	45	477
BA120226	47	Twigs	N/A	265	30	47
BA120228	228	Twigs	N/A	1810	30	228
BA120229	300	Twigs	N/A	3130	30	300
BA120230	377	Twigs	N/A	5105	25	377
LZU045**	377	Bulk organic		6620	40	377
BA120231	411	Twigs	N/A	5785	25	411
BA120241	437	Twigs	N/A	6715	35	437
BA120232	477	Twigs	N/A	7535	30	477
LZU046**	477	Bulk organic	N/A	9670	40	477
Beta333693	485	Twigs	-28.5	7720	40	485

* All core depths are converted to the depth of core XY08A

** To make sure the calibration of the two cores are corrected, we also used two measured Bulk organic samples from core XY08A, the two AMS data match well with core XH-14-X-94's curve within permissible error range

## Results

Core XY08A was drilled from the central part of the lake. Since the wood twigs we used for dating materials were all small and intact, they were deemed unlikely to have been re-worked from earlier initial deposition. Therefore, our chronology profilesfrom core XY08A were reliable. However, in the case of original core XH-14-X-94 [2], only bulk sediments were chosen for radiocarbon dating without consideration for any potential reservoir effect. Thus, by calculating the difference in ^14^C ages between core XY08A (this study) and core XH-14-X-94, the reservoir effect in Lake Xingyun can be evaluated.

**Fig 3:**
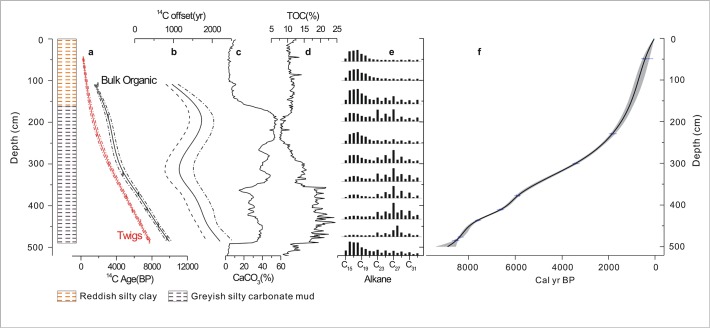
Lithology, radiocarbon results, proxies and age-depth model of core XY08A from Lake Xingyun. (a) Radiocarbon age-depth model for Xingyun lake using twigs (red) and bulk organic material (black). (b) Measured and modeled reservoir age offset for Lake Xingyun. LOI (c) and carbonate content (d), (e) *n*-alkane record of Lake Xingyun. Relative abundance (vertical) versus carbon distribution (horizontal) of *n*-alkane homologues in Core XY08A. The carbon number distribution is from C15 to C33. (f) The age model (solid line) using the method of Heegaard *et al*. [43]. The shaded envelope indicates the 95% confidence interval.

To ensure a direct comparison between these two cores, we converted the depth of XH-14-X-94 to that of XY08A by directly comparing their carbonate content (Fig. 2). For additional support for these correlations, we also used two measured bulk organic samples from core XY08A. The AMS data from 2 bulk samples in core XY08A match well with the data from core XH-14-X-94 within the permissible error range (Table 1, marked with **). The correlation below 4.9 m of the XY08A core and 8.2 m of the XH-14-X-94 core was not performed, as no significant changes in carbonate content were observed for the lower part of the cores (Fig. 2). Because the radiocarbon ages from the two cores were not from the same depths, we used the mixed-effect regression model based on the work of Heegaard *et al*. [43] by fitting a cubic spline function of smoothing through the 68% age ranges and assigning a 95% confidence level to the interpolated ages at 2 cm intervals. As such, two successive radiocarbon age-depth curves were obtained (Fig. 3A, B), which represent the bulk organic matter and plant macrofossil ages, respectively. The radiocarbon reservoir offset (*RRO*) was determined by calculating the difference between the un-calibrated conventional radiocarbon ages of the bulk organic (*RBO*) and the plant macrofossils (*RPM*) at equivalent depths (Fig. 3C). The equation for the uncertainty in the *RRO* was derived from the propagation of errors, assuming independence between *RBO* and *RPM* was calculated as: $\sigma_{RRO}=\sqrt{\sigma_{RBO}^{2}+\sigma_{RPM}^{2}}$ where *σ*_*RBO*_ and *σ*_*RPM*_ were the uncertainty in *RBO* and *RPM*, respectively.

Due to the lack of radiocarbon data above the depth of 109 cm from either core XH-14-X-94 or core XY08A, we only focused on the calculation of the radiocarbon reservoir offsets between the depths of 490–109 cm. As shown in Fig. 2, all the AMS ages on the bulk organic matter were significantly older than those of the twigs. The data suggested that the TOC chronology contains carbon depleted in ^14^C with respect to the atmosphere at the time of deposition, consequently yielding older ages. The differences between these two chronological profiles varied between 960 and 2200 years at years between depths of 490 and 109 cm.

## Discussion

### 1. Influence of lake hydrology on changes in reservoir effect in Lake Xingyun

The radiocarbon reservoir offset can be attributed to many factors. Some of the reservoir effect is derived from the hard water effect (HWE), which is related to riverine runoff containing "dead" dissolved inorganic carbon [44]. The reservoir offset can also be derived from fully aquatic plants (submerged macrophytes and phytoplankton algae), which assimilate dissolved inorganic carbon (DIC) in lake water containing depleted ^14^C compared to the atmosphere (the "lake-carbon reservoir" effect). On the other hand, reworked organic materials from catchment weathering [26] can dilute the ^14^C content of organic materials with respect to the atmosphere at the time of deposition, causing the apparent ages to be older than the depositional event [45]. In the following section, we compare the radiocarbon reservoir ages of Lake Xingyun with the LOI, CaCO_3_ content and *n*-alkane distribution (Fig. 3) to better understand the origin of the radiocarbon reservoir effect in Lake Xingyun. Based on these findings, we further qualitatively explain how the regional climate might affect the basin's reservoir age by influencing lake water budget and riverine runoff as well as bedrock weathering.

Organic matter derives from terrestrial plants, aquatic plants, and reworked organic matter washed into the lake from the shore due to strong hydrological dynamics. Thus, bulk sediments are likely to be a mixture of autochthonous (from phytoplankton) and allochthonous (from terrestrial plants) organic matter with the proportion of each varying through time [14], depending on the lake levels and aquatic productivity. On the other hand, carbonate content can be used as a complementary indicator of hydrological balance [46, 47]. However, the increase in carbonate and organic matter input could also be due to changes in lake size, either through lake shrinkage or expansion. Therefore, in our study, we have also introduced the *n*-alkane record to further examine differences in organic matter from terrestrial compared with aquatic sources. The dominant chain length of the *n*-alkanes mainly depends on the source organisms. Generally, the distribution of *n*-alkanes from Lake Xingyun can be divided into two main categories: one is dominated by the C_27_-C_31_ components, which are typically derived from the leaf wax of vascular plants [48–52]; the other is dominated by C_15_-C_19_ homologues contributed by algae and bacterial sources [49, 53, 54]. The predominance of terrestrial higher plants, including moss, sedge, and lichen may lead to the average chain length (ACL) values of the *n*-alkanes, while an increased input of aquatic macrophytes may lead to a lower ACL value. Although different species of plants may influence the ACL index, preliminary data from pollen and non-pollen palynomorphs [55] indicate that Holocene vegetation has not changed dramatically. If so, the ACL index is primarily influenced by the proportion of aquatic to terrestrial plants.

The radiocarbon reservoir effect of Lake Xingyun would be stationary, if the hydrological conditions remain the same. Since the radiocarbon reservoir age changes with time, this suggests that the hydrological conditions have also altered. Therefore, we discuss the radiocarbon reservoir effect in the following three intervals: 4.9–3.2 m, 3.2–2.0 m and 2.0–1.0 m. Generally, the reservoir offset decreased between depths of 4.9–3.2 m and 2.0–1.0 m and increased at 3.2–2.0 m, suggesting that Lake Xingyun underwent changing hydrological conditions through time.

#### 1) 4.9–3.2 m, decreasing reservoir effect

Below 4.9 m, TOC and carbonate contents were extremely low. The *n*-alkane results showed a predominance of C_15_-C_19_, which were potentially contributed by algae and bacteria. Between 4.9 and 3.2 m depth, the TOC content increased significantly, suggesting that there was a higher organic input, possibly due to hydrological changes such as the formation of closed lake conditions. The *n*-alkane results suggest that there was an increase in terrestrial material relative to aquatic sources, as determined by the domination of long chain *n*-alkanes (C_27–31_). The high carbonate content at this section may indicate low lake levels and a hydrologically closed basin. Therefore, we suggest that more terrestrial organic matter was delivered into the lake center, diluting the existing aquatic carbon pool.

#### 2) 3.2–2.0 m, increasing reservoir effect

In this section, we observed increased carbonate content but decreased TOC values. Based on the *n*-alkane results, the organic composition changed from mixed terrestrial and aquatic plants to mainly aquatic plants. This change suggests that lake level increased gradually and reached the highest stand at the top of this section. Under such conditions, terrestrial organic matter cannot be easily transported to the lake center where the sediments mainly originated from the deposition of authigenic carbonate and microphytic algae, resulting in increased carbonate levels and decreased TOC in the core. Therefore, the increased radiocarbon reservoir age should be mainly related to the HWE.

#### 3) 2–1 m, decreasing reservoir effect

In this section, TOC increased and remained at the median value, while carbonate decreased significantly to nearly zero. Based on the *n*-alkane results, the organic matter in this section was mainly derived from aquatic algae sources, with occasional macrophyte inputs. Therefore, Lake Xingyun was probably at a high lake level. In this case, the terrestrial organic materials barely reached the central lake coring site, and the decreased radiocarbon reservoir age was probably not caused by dilution with terrestrial organic matter. Therefore, we suggest that the diminished hard water effect at this stage may be due to a change from a closed to an open lake system at this time.

In summary, over the interval of 4.9–2 m sediment depth, the variation in the radiocarbon reservoir ages in Lake Xingyun was mainly caused by the introduction of contemporaneous organic matter with variable amounts of old terrestrial organic matter flushed into the lake from the catchment. However, at the depth of 2–1 m, the decreased radiocarbon reservoir age was mainly controlled by the *in-situ* hard water effect, which likely reflects a change in the lake status from a closed to an open system. These results provide further insight into the hydrological conditions and terrestrial input in Lake Xingyun during the Holocene.

### 2. Paleoclimatic implications of the revised records from Lake Xingyun

**Fig 4:**
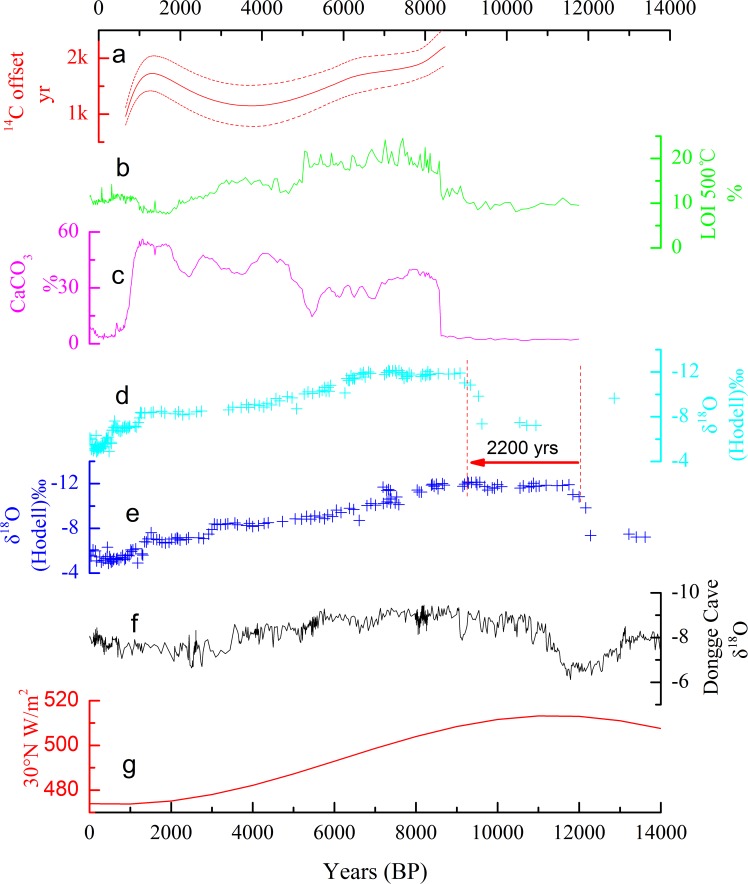
Comparison of various climate proxies in the Holocene. (a) Changes in radiocarbon reservoir age during the Holocene; LOI (b) and carbonate content (c) of Lake Xingyun for this study; changes of bulk carbonate oxygen isotope (d) during the Holocene from Hodell *et al*. [2], conversion of bulk organic radiocarbon chronology to terrestrial twigs AMS dating radiocarbon chronology; changes of bulk carbonate oxygen isotope (e) during the Holocene from Hodell *et al*. [2]; (f) δ^18^O record from Dongge cave [57]; (g) June insolation at 30°N [58].

When comparing our radiocarbon chronology with that of Hodell *et al*. [2], our data suggest a different interpretation of the previously published proxy data from Lake Xingyun. Based on our results, the cold event corresponding to YD in NW Europe event identified by Hodell *et al*. [2] from their δ^18^O record may have actually occurred during the early-middle Holocene (Fig. 4). If the bulk inorganic ^18^O indicates the strength of the ISM, then the ISM would not have been intensified until 9–8.5 ka, which is in contrast to the records of monsoons in the neighboring region [56]. This suggests that the ^18^O record from Lake Xingyun might not be a proxy of monsoon intensity. Rather, it might mainly be related to carbonate precipitation. Furthermore, our study highlights the significance of an accurate chronological profile for the valid interpretation of proxy records, since most proxies can be interpreted in various ways. Another critical issue identified in this study is that the highest productivity of Lake Xingyun occurred at approximately 8 ka (Early to Middle Holocene transition) instead of approximately 12 ka (glacial-interglacial transition). Such change in productivity might result from the regional hydrological setting instead of large-scale monsoonal activities. More studies now become necessary in this region to scrutinize how monsoon strength affected the hydrological condition of this lake.

## Conclusions

1. There exists a pervasive reservoir age in Lake Xingyun during the last 8500 years which changes between 1150 to 2200 years since the Early Holocene.
2. Changes in reservoir age are associated with inputs of either pre-aged organic carbon or ^14^C-depleted hard water in Lake Xingyun, which were likely caused by hydrologic changes in the lake system.
3. The radiocarbon reservoir offset can be used as an indicator of the carbon source in the lake ecosystem and depositional environment. This effect can also provide useful insights for land use in the catchment basin around a lake.
